# Sustained remission of Multicentric Castleman Disease in children treated with tocilizumab

**DOI:** 10.1186/1546-0096-9-S1-P6

**Published:** 2011-09-14

**Authors:** C Galeotti, A Boucheron, S Guillaume, I Koné-Paut

**Affiliations:** 1 Department of Pediatric Rheumatology, CEREMAI, CHU Bicêtre, University of Paris Sud, France

## Background

Multicentric Castleman Disease (MCD) is an idiopathic lymphoproliferative disorder, exceptionally reported in children, probably due to an increase of interleukin 6 secretion. MCD is characterized by systemic lymphadenopathy and constitutional inflammatory symptoms including severe growth retardation. Previous studies in adult showed that anti-interleukin 6 receptor antibody alleviated symptoms and biochemical abnormalities of MCD.

## Aim

We describe efficacy and safety of tocilizumab (TCZ) in two children with MCD. Moreover we describe the long-term effect and dose adjustments in one of them with sustained remission after 3 years.

## Methods

MCD was suspected clinically then confirmed histologically in both cases. Tocilizumab was administered intravenously at a dose of 8 mg/kg every 2 weeks.

## Results

TCZ treatment alleviated fever and restored growth velocity in both patients. In parallel biochemical abnormalities, including hypergammaglobulinemia, increased C-reactive protein (CRP) and erythrocyte sedimentation rate (ESR) returned to normal ranges. For the first patient, the size of abdominal lymph nodes decreased but the splenomegaly persisted. For the second patient, the multiple profound adenopathies persisted and a hepatic node of 7 mm appeared. The side effects in both cases were mild but sustained thrombocytopenia. Infusions intervals were spaced after 10 months for the first patient who had sustained remission since 3 years. The second patient is treated every two weeks since 8 months.

## Conclusions

TCZ is effective and safe in children with MCD. The redundant effect of the drug allows infusion adjustments on a long term-administration. However our observations cannot confirm that TCZ may cure this serious disease.

